# Multiscale Enaction Model (MEM): the case of complexity and “context-sensitivity” in vision

**DOI:** 10.3389/fpsyg.2014.01425

**Published:** 2014-12-19

**Authors:** Éric Laurent

**Affiliations:** ^1^Laboratoire de Psychologie EA 3188, Unité de Formation et de Recherche Sciences du Langage de l’Homme et de la Société, University of Franche-ComtéBesançon, France; ^2^Maison des Sciences de l’Homme et de l’Environnement Claude Nicolas Ledoux, UMSR 3124, CNRS and University of Franche-ComtéBesançon, France

**Keywords:** autopoiesis, distributed cognition, dynamical systems, embodied cognition, embodiment, enactivism, motivated perception, situated cognition

## Abstract

I review the data on human visual perception that reveal the critical role played by *non-visual* contextual factors influencing visual activity. The global perspective that progressively emerges reveals that vision is sensitive to multiple couplings with other systems whose nature and levels of abstraction in science are highly variable. Contrary to some views where vision is immersed in modular hard-wired modules, rather independent from higher-level or other non-cognitive processes, converging data gathered in this article suggest that visual perception can be theorized in the larger *context* of biological, physical, and social systems with which it is coupled, and through which it is enacted. Therefore, any attempt to model complexity and multiscale couplings, or to develop a complex synthesis in the fields of mind, brain, and behavior, shall involve a systematic empirical study of both connectedness between systems or subsystems, and the embodied, multiscale and flexible teleology of subsystems. The conceptual model (Multiscale Enaction Model [MEM]) that is introduced in this paper finally relates empirical evidence gathered from psychology to biocomputational data concerning the human brain. Both psychological and biocomputational descriptions of MEM are proposed in order to help fill in the gap between scales of scientific analysis and to provide an account for both the autopoiesis-driven search for information, and emerging perception.

*“Es ist nicht zu leugnen, daβ* auf die Dauer* über jeden Einzelnen dieser großen Zwecklehrer bisher das Lachen und die Vernunft und die Natur Herr geworden ist: die kurze Tragödie ging schließlich immer in die ewige Komödie des Daseins über und zurück, und die “Wellen unzähligen Gelächters” — mit Aeschylus zu reden — müssen zuletzt auch über den größten dieser Tragöden noch hinwegschlagen ^1^.”*

Friedrich Wilhelm Nietzsche, 1882.

Die fröhliche Wissenschaft.

Erstes Buch, 1, “Die Lehrer vom Zwecke des Daseins.”

## INTRODUCTION

This paper deals with complexity in *vision*. Its purpose is to examine how conceiving complexity – in this specific case – implies to generate (largely generic) intellectual tools, which allow gathering and *synthesizing* data emerging from mind, brain and behavioral sciences.

From a behavioral point view, as well as from an anatomical-physiological point of view, vision can be studied in relation to non-visual processes (e.g., basic homeostatic loops, emotions, higher-level cognition, pathological events, social-economical factors). Here, I am presenting a perspective on *vision* that builds on previous work on enactive (Varela et al., 1991; Thompson, 2010; see also Stewart et al., 2010; Di Paolo and De Jaegher, 2012; McGann et al., 2013, for a view on the diversity of recent enactive research), embodied (Clark, 1999; Chemero, 2001, 2009; Thompson and Varela, 2001) *and* situated (Clark, 1997; Noë, 2009) cognition trends, empirical data in experimental psychology, works in theoretical integrative biology (von Bertalanffy, 1951; Weiss, 1963; Chauvet, 1993a,b,c, 2004; Kozma et al., 2005), and recent research in the neuroscience of brain connectivity. I focus the work on the various ecologies in which vision is embedded, *including the organism’s subsystems demands*. The contexts in which biological vision emerge are multiple, and can be described at different scales. At this point of science development, these scales range at least from the cellular level to the social-economical level. Theoretical and empirical data suggest the need to consider *multilayered context* as a predictor of behavioral and phenomenological activities of vision systems. In contrast with some previous views on enaction, I promote the principle of bottom-up teleological influences that weigh on visuomotor processes – in order to integrate the various couplings between biological processes.

The basic properties of the Multiscale Enaction Model (MEM) are conceived from the principles of emergence. As opposed to many approaches to psychology research, this model identifies multilayered contexts, which co-constitute mental processes. Similar general reasoning has been applied to theoretical biology. As reported by Weiss (1963, p. 389), the analytical process by which a cell is decomposed into its constituent atoms and ions does not provide much information about the difference between a live and a dead cell: “In trying to derive the more complex systems from their elements, therefore, one must make up for this deprivation somehow by restoring the lost properties. The practice of doing this through symbols, such as ‘organization’ or ‘integration,’ is an old one, but seldom makes explicit as to whether these symbols are meant to be final logical postulates to compensate for the limitations of pure reductionism or merely provisional promissory notes that they will ultimately yield to analytical resolution.” Because the problem’s essence of cellular unity is tightly linked to “the indispensable *cooperative existence of all these features”* (i.e., genic replication, contractility, respiration, selective permeability …). In the same vein, the problem of organization in psychology has often been conceived at an abstract cognitive/psychic level, through the intervention of “executive functions” and different “memory,” representational systems. The thesis underlying the conception of MEM is that the mainstream tendency consisting in evoking *dei-ex-machina* as the *loci* of control of behavior (see Bruner and Goodman, 1947, for early critics of this tendency) conducts to consider the functioning “laws” of cognitive “structures” or “functions” as the central task of cognitive psychology, whereas these “structures” have been inferred in specific contexts, yielding specific couplings within the individual and between the individual and the external environment. This process is associated to both a foundational tunnel vision and amnesia. The tunnel vision refers to the neglect of complexity and multiscale connectedness. Complexity generates cognitive and behavioral variability at the macroscale. Instead of seriously considering this complexity and coordinative processes at a more elementary level, the privileged process is to conceive centralized structures of control and to define variability as error (in relation to the “standard” behavior initially modeled). Then, the amnesia refers to the forgetting process of the foundational context, in which the “rules” or “laws” of cognitive structures were inferred. Progressively, “structures” and “functions” get increasingly autonomous from other elementary connections at a lower level.

The central feature of MEM is its sensitivity to multi-layered context. I define the “context” of any biological unit of interest as any other elementary (or groups of) living or non-living material or symbolic objects, which, through their connectedness with the first biological unit, may influence its activity. Hence, there is an acquaintance relation between MEM and previous embodied (i.e., accent put on the bodily context), enactive (i.e., special interest in the sensorimotor contingencies, and sometimes in the autopoietic process), and situated (i.e., inseparability between cognitive processes and their context of production, the latter being conceived at different scales as a function of the authors) approaches. These approaches all have different interests. However, the originality of MEM also lies in the *flexibility* that accompanies the scale level attached to the considered context. The multiscale character of the model lies in its ability to connect perceptual activity to a wide range of contextual influences, whose level of abstraction can considerably vary.

The procedure that is employed in this article consists in gathering empirical evidence for promoting MEM as a unifying paradigm within psychological science. A discussion of its detailed properties as well as its links with theoretical integrative biology models is proposed in the final part of the paper.

In the following lines, I successively review seminal and foundational approaches to dynamics and systemism in visual perception, gather recent empirical works that implicitly or explicitly give *evidence for multiscale couplings* between visual behaviors (or phenomenology) and various other systems, and report neuroscience findings that inform us about how brain networks support integrative cognitive processing. Finally, I summarize salient features of MEM: multiple connectedness, embodied, multiscale, flexible teleology, as well as emergent and dynamic operational couplings. Potential impacts of MEM on both a synthesis in the field of brain and mind sciences, and psychological intervention in complex and real settings are discussed.

## THE CHALLENGING VIEWS OF EMBODIED AND CONTEXT-SENSITIVE VISUAL PERCEPTION

There has been a long history of exchanges between the fields of psychology, biology and computer science. These relationships have influenced the way cognitive psychologists themselves have considered perceptual and cognitive abilities. In the classical and radical computational-symbolic approach to human cognition, which heavily relies on mental representations, visual-perceptual information is ambiguous and requires further cognitive interpretation and enrichment from the observer. In this framework, the impenetrability of perception (Pylyshyn, 1999) is explicitly or implicitly established. On the explicit side, some authors defend hypotheses such as the informational encapsulation of early vision (Raftopoulos, 2001). The data that are discussed throughout the current paper, in contrast, highlight the need to build a theory in which visual processes are seen as coordinated and emerging phenomena. I want to present existing data on humans that show how vision is coordinated with other systems and to take the case of vision as an opportunity to develop a proposal for a ‘modern synthesis’ in the sciences of mind. Thanks to the coordinative property of mental and neural nets, vision systems behave as real intelligent systems by continuously adapting to the current goals of the agent.

### A PATH TOWARD SYNTHESIS: FIRST SYSTEMIC APPROACHES IN PERCEPTUAL PSYCHOLOGY

Over the last few decades, many disciplines in science have been increasingly concerned with the consideration of complexity and dynamical non-linear phenomena (Capra, 1997; see Beer, 2000, for the case of cognitive science). This has also been the case of psychological approaches to vision. In cognitive psychology, one of the first trends concerned with the consideration of the dynamic nature of visual perception was the “New Look” stream, whose prominent figures were Jerome S. Bruner and Leo Postman (see Bruner and Postman, 1949, for an overview). The basic idea developed in the 1940s was that visual perception in humans was connected to other psychological systems that constitute the observer’s global personality. Building on the statement that perception should not be studied independently from “the rest of the dynamical system that constitutes the person,” Bruner and Goodman (1947, p. 33) claimed what the psychological study of perception should be: “the problem is, indeed, to understand how the process of perception is affected by other concurrent mental functions and how these functions in their turn are affected by the operation of perceptual processes.” This was clearly a strong proposal characterized by the systemic concern developed by the authors. At the time, many other approaches, actually considered the perceiver as if it was a rather passive recording instrument. “One might, in most experiments, describe him in much the same graphical terms as one uses to describe the latest piece of recording apparatus obtainable from Stoelting or the American Optical Company. Such psychology, practiced as it were *in vitro*, has fallen short of clarifying the nature of *perception* in everyday life much as did the old nerve-muscle psychophysiology fall short of explaining *behavior* in everyday life” (Bruner and Goodman, 1947, p. 33). What the authors criticized in their paper was also the discrepancy between the lab situation and the marketplace. In everyday life, many factors interact and change the perception that emerges. In order to understand those changes, we must give credit to variability and analyze its underlying mechanisms. One major obstacle to the development of the approach of the authors in the years following their publication was probably the relationship of a lot of researchers to variability, which was often considered as noise or as being associated to the effects of “attention.” Bruner and Goodman (1947) also questioned the latter concept, which usually prevents researchers from getting further into the causes of variability, and especially into the description of the relationships between perception and other systems. That is, by invoking a *deus-ex-machina*, one does not make significant progress in the description of *what* system really dynamizes perceptual activity. By invoking attention as a cognitive structure, perceptual psychologists sometimes avoid considering perception as an open system, or to be more precise, avoid considering that perception *forms* systems with non-perceptual processes.

Bruner and Goodman (1947) investigate perceptual processes in the context of social-economical needs. In one of their tasks, they asked poor and rich children from Boston to evaluate the size of coins. Participants had to manipulate a knob, which controlled the diameter of a projected circle of light. When they judged that the diameter of the projected circle was equivalent to the one of the coin they had in the palm of the left hand – at the level of the light, and six inches to its left – then the trial was over. Results showed that all the participants overestimated the size of coins, but this overestimation was more important among the poorer than among the richer children. Interestingly, other children took part in the experiment in the “control condition” in which coins were replaced by medium-gray cardboard disks of identical size. In this condition, no overestimation was reported. The perception of similarity of sizes between two objects that are simultaneously available in the visual field is *influenced* by both the financial needs of participants and the value of stimulation. The variability of visual similarity perception across participants can be understood if we consider perception in the broader context of social-economical factors. That is, the emergence of visual perception is dependent upon *the initial conditions of the perceiver*. The sensitivity to initial conditions is a basic property of dynamical systems that will be discussed later on in this paper. In order to better understand the evolution of those initial conditions and their potential impact on the emergence of visual perception, I will go on with the review of systemic vision models. Understanding sensitivity to initial conditions implies to develop *a conception of the connectedness of perception to other systems*. In the next paragraphs, I build on the discussion of different systemic trends in order to provide a *synthetic conception* of the place of perception within the living organism.

### THE GIBSONIAN ECOLOGICAL APPROACH TO VISUAL PERCEPTION: IDENTIFICATION AND CRITICS

One other major approach in the systemic views landscape is ecological psychology. Gibson (1950, 1966, 1979) proposed to study perception as an embodied process that couples with motor action. He assumed that we could not understand perceptual adaptation to the world if we isolate perceptual activity from motor abilities and actions. The systemic nature of the approach lies in the tight association between perception and action. Gibson’s research seems to be founded on basic postulates: (1) the human subject and the environment should be modeled in their reciprocal information; (2) relevant information is made of “invariant” optical elements and gradients (of texture, speed …); (3) perception – a pick-up process of (environmental) optical invariants – informs us about our relation with the environment and is directly “meaningful” in terms of potential actions (i.e., affordances). The systemic nature of the approach is well illustrated in the description of action-dependent perceptual invariants. For instance, as a function of your heading direction, the nature of visual flow will qualitatively vary. More specifically, the focus of expansion (FOE) – which is the optical point from which a radial pattern of velocity vectors develops – evolves in the visual field as a function of heading direction. The FOE signals to the observer the current heading direction. If the observer’s movement changes such that the heading direction also changes, then the place of the FOE changes accordingly. Action creates information. Here, we realize that perception must be understood in relationship to action. Gibson proposes *a synthesis* between perception and action in order to predict both perceptual and motor activity as a function of each other. “We must perceive in order to move, but we must also move in order to perceive” (Gibson, 1979, p. 223). The observer’s ability to detect this kind of change (i.e., the position of the FOE) is allowed by the intrinsic nature of sensory systems, which are formatted to be sensitive to gradients of velocity.

One of the most advanced steps in recent ecological studies is the production of “laws of control” which mathematically bind perceptual information and motor parameters in order to give an account for the way humans control movement, on the basis on perceptual information. This has been accomplished by unifying the fields of ecological psychology and dynamic systems theories (Kugler and Turvey, 1987; Lee, 1998; Warren and Fajen, 2004). For instance, Warren et al. (1986) modeled human motor regulation of running over an irregular surface. The task constraints implied that participants adjust step length as a function of the demands of the irregular terrain. The authors showed that motor action could be regulated on the basis of the available visual data in the optical flow, which was created by the runner’s motion. While approaching the irregularities of the terrain, the observer experiences the optical expansion of those objects. The time-to-contact (tau or τ; Lee, 1980) can be extracted from this cue, since the time-to-contact is given by the inverse of the relative rate of visual object dilation. Furthermore, Warren et al. (1986) demonstrated in their study, that for adjusting step length as a function of irregularities, participants could adjust step duration as a function of vertical impulse (I) [given that mass (m) and gravity (g) are quasi-constant] by using optical information derived from τ, that is, Δτ, which is the difference in time-to-contact between two surfaces:

I=m.g.Δτ⁢                        (1)

This kind of control-law illustrates well the very strong coupling between the current movement and available visual information, and that critical information for subsequent coordination of movements can be extracted from this dynamical change in visual stimulation. Information is available in the pattern of change of stimulation as a function of time, so that vision is non-dissociable from and – in our own terms applied to those data – *enacted by* the current motor context of the organism.

More recent empirical research using virtual reality in humans, such as the study by Warren et al. (2001), have also proposed some kinds of elaborate control-laws, for instance to explain how humans guide locomotion to a goal. In this type of law, flow information is combined to another *visual* information, which is the egocentric direction of the visual goal. The reported control-law is a linear combination of flow and of the perceived direction of the visual target, weighted by the magnitude of flow (Warren et al., 2001). Among recent developments, and beyond modeling of human behavior and applications in neural networks, ecological psychology has also found an allied in behavior-based robotics (Arkin, 1998), specifically in a Gibsonian trend (Duchon and Warren, 1994, 2002) sometimes so-called “ecological robotics” (Arkin et al., 1998; Duchon et al., 1998). For instance, Duchon and Warren (1994) noted that laws of control were applicable to any moving agent (see, for recent instantiations of the principle in the critical context of autonomous airborne navigation, Serres et al., 2006; Franceschini et al., 2007). In their paper, Duchon and Warren (1994) proposed two laws of control that they tested on an actual robot that evolved in an unmodified office environment. The laws concerned the obstacle-avoidance problem. Building on previous studies conducted by Gibson in humans on the one hand, and by Srinivasan and Gregory (1992) in insects on the other hand, the authors proposed to implement their two laws of control, which respectively corresponded to the Balance Strategy and to the Avoid-Closest Strategy. The first one acts to equate the rate of optic flow in the left and right halves of the visual field, whereas the second one, inspired from the previously presented *tau* variable, makes the agent turn from the place of the visual field with the lowest time-to-contact. They reported that, globally, their robot succeeded well in avoiding obstacles while moving in a real environment.

However, a major limitation of the Gibsonian ecological approach, especially in psychology where humans are modeled, lies in the reduction of the system in which vision is embedded, to a two-dimensional (perception-action) scheme. Though the central point of this paper concerns the systems in which vision is hypothesized to be contextualized, the direct character of perception in Gibson’s approach should be shortly discussed here. Information, according to the ecological approach, is conceived as being unambiguous and specifying directly affordances, which are perceived opportunities of action in a given environment, and given the biological properties of the organism. Those biological properties are related to the ones that underlie opportunities of motor action (e.g., the height of the leg, which is reported to the height of a stair in order to determine whether the latter is ‘climbable’; Warren, 1984). The environment is processed and measured in relative units, as a function of biomechanical and physiological properties of the perceiving organism. What I want to defend here is that this is just one single kind of embodied and context-sensitive vision. Nevertheless, vision is not only sensitive to the motor properties of the organism. Although the Gibsonian ecological approach is systemic, it has a “single-scale” focus of analysis. I would rather suggest adopting a “multiscale” approach to context-sensitivity. In order to develop this view, I will provide the reader with examples related to different *scale levels* in the analysis of contextual influences.

## ZOOM OUT! COGNITIVE AND BEHAVIORAL FOUNDATIONS OF THE MULTISCALE ENACTION MODEL

This multidimensional view on the determinants of vision relies on the acknowledgment of the complex interplay between vision and *non-visual factors*. Therefore, I will review empirical studies in humans showing the influence of basic appetitive drives, biomechanical constraints and fatigue, mood and affective processes, higher-level cognition and cognitive expertise. These influences will be studied as a function of their type (i.e., an impact on phenomenological experience *per se*, or an early influence on the orientation of visual sensors). Each factor of influence will be considered as a contextual parameter that should not be neglected, when one wants to understand and/or influence human perception.

### THE SEE-WHAT-YOU-NEED EFFECTS: BASIC DRIVES MODIFY SENSITIVITY TO VISUAL STIMULATION

Some relations between basic drives and perception have been studied in the field of neuroscience through the concept of alliesthesia (Cabanac, 1971, 2006), showing that hedonicity was a central component in behavioral regulation. At the sensory level, the same individual differently evaluates a given stimulation as a function of his internal equilibrium, and according to the principle that what is pleasant is what is useful. Therefore, while the state of the organism is evolving, the hedonic relation to a given stimulation simultaneously evolves. This phenomenon has been known as alliesthesia under the influence of Cabanac.

More specifically, in the field of vision, Changizi and Hall (2001) proposed to test the differential effects of salt and water ingestion before a judgment task where the transparency of different categories of stimuli had to be evaluated. Immediately before the judgment task, the 37 participants distributed in the thirsty group ate one lunch bag of salty chips (35 g, 190 kcal, 350 mg sodium) whereas the 37 participants distributed in the non-thirsty group were supposed to drink water until not thirsty. Stimuli were then presented through a stereoscope. Three categories of stimuli were tested (i.e., stimulus with high probability that it is due to a scene with a transparent surface: “definitely transparent”; ambiguous stimulus for which probability that there is a transparent surface is neither very high nor very low: “ambiguously transparent”; stimulus with very low probability that it is caused by a scene with a transparent surface: “definitely not transparent.” Participants used a computer mouse to press a “transparent” button if they perceived a transparent surface, and to press a “not transparent” button if they did not perceive a transparent surface. Results showed that the experimental ingestion had a significant effect for the “ambiguously transparent” stimuli. Participants belonging to the “thirsty group” exhibited a greater inclination to perceive transparency than the participants belonging to the “non-thirsty group.” The authors’ theoretical framework is both probabilistic and utilitarian. Probabilistic because the derived percept is hypothesized to be the best “bet” on the basis of available stimulation, and utilitarian because the best “bet” depends not only from what is most probable, but also from the costs and benefits of the stimulation for the organism. Changizi and Hall (2001) interpret their experimental data, stating that salt ingestion conducted to change the utilities attached to visual stimulation and that thirsty participants tended to be more sensitive to transparency because that is a typical visual property of water; and water is the element needed by thirsty participants. In our terms, visual processing would embody basic needs in order to satisfy the current goals of the organism. This shows the need to develop our understanding of motivational influences over vision and visual judgment (see also Balcetis and Dunning, 2006, 2010, for a complementary view of such influences).

Visual judgment, when considered at the psychological level, is highly dependent on what occurs at the physiological level. If psychological performances on the one hand, and physiological loops involved in hydration regulation on the other hand, are not considered as coordinated parameters, and if they are not conceived synthetically, the meaning of cognitive judgment does not emerge. In the latter case, there is an epistemological gap between cognitive processes, which are described as a sequence of decontextualized operations, and the rest of the body to which they actually refer.

### CARRYING HEAVY LOADS AS WELL AS FATIGUE MAKE YOU OVERESTIMATE HILL INCLINATION

Other authors have also proposed a framework providing insights into how visual information processing in humans can be influenced by internal dynamical factors. Proffitt (2006) and his Group, from the University of Virginia, developed a theoretical framework, as well as an experimental program on the impact of observer’s physical potential on visual judgment. According to Proffitt (2006, p. 110), “[v]isual perception is not solely a visual process. What one sees in the world is influenced not only by optical and ocular-motor information, but also by one’s purposes, physiological state, and emotions. Perceptions are embodied; they relate body and goals to the opportunities and costs of acting in the environment.” According to the researchers, perception and judgment would express the opportunities of acting in the environment. Instead of “coldly” and stably judging physical values of environmental dimensions, we would do it as if we were intending to physically act in the environment. In one of their classical experimental situation, participants are asked to judge the inclination of small hills. Proffitt et al. (1995) investigated the ability of humans to estimate the inclination of those hills as a function of three response modalities. Individuals were either required to answer verbally, or they would give their response by visually aligning a disk such that the inclination of a part of the disk was equivalent to the inclination of the hill, or they would manipulate a haptic device (i.e., a palmboard) without controlling the device visually. The authors found that the participants generally overestimated hill inclination in both verbal and visual modalities but not in the haptic one. Estimations affected by overestimation concern measures of “explicit awareness,” according to the authors. Explicit overestimation of slant would be useful because it would promote “a heightened sensitivity to differences in the small inclines that people can actually traverse” (see Proffitt, 2006, p. 113). The position is evolutionary in nature. The eye probably integrated evolutionary pressures concerning not only optics but also other factors that would express the economy of action and the subjective value associated to acting in a particular environment. In this vein, Proffitt et al. (1995) similarly demonstrated that physical exhaustion changed the amplitude of slant overestimation. In one experiment, they recruited regular runners and asked them to evaluate different hills’ inclination before and after difficult run training. In both visual and verbal modalities, the angle judged after running was significantly higher than before running. This pattern of results suggests that participants visually and verbally evaluate inclination as if they were projecting to physically move toward the summit. Here, the physiological context of the organism leads to biased visual judgment as a function of motor availability of participants. This has been confirmed since by other studies showing that wearing a heavy backpack lead to the same types of overestimations (Bhalla and Proffitt, 1999).

What this kind of data suggests is that visual judgment is fundamentally context-sensitive, and this context can be, among others, the biomechanical constraints as well as the current physiological state of fitness of living organisms.

### ARE YOU HAPPY? THEN YOU ARE READY TO CLIMB THE HILL AND TO SEE THE GLOBAL PICTURE!

In a more recent study, Riener et al. (2011) manipulated participants’ mood and placed them subsequently in the previously presented protocol of slant evaluation. Further evidence for an embodied perception of spatial environment was provided. Participants in a sad mood reported hills to be steeper. Riener et al. (2011) interpret those data in terms of energetic potential, which leads to anticipate more or less subjective cost associated to climbing the hill.

Other protocols investigate the impact of mood on the variation of similarity judgments of hierarchical (compound) stimuli. Building on a psychophysical-like task proposed by Kimchi and Palmer (1982, global-local focus test), Gasper and Clore (2002), in their second experiment, investigated the relationships between mood induction and the weighting of global and local factors of visual similarity. After inducing respectively happy, neutral, and sad moods in three different experimental groups (using a writing procedure of autobiographical events), they asked participants to perform the similarity judgment task. In this task, three compound stimuli were simultaneously presented: one at the center above the two others, which were located respectively on the left and on the right of the display. Participants were supposed to decide which, among the two figures presented below, was the more similar to the above presented shape (i.e., reference shape). One of the two options was globally similar (e.g., globally a triangle) to the reference shape, and the other one was locally similar (e.g., made of small squares) to the reference shape. Results showed that participants in negative mood were less likely than individuals in a positive or neutral mood to use the global form as a basis for matching the objects. The experiment therefore indicates that visual similarity judgment is influenced by mood state.

According to Clore and Huntsinger (2007) mood moderate natural or spontaneous tendency. Positive mood would correspond to a “GO” signal and negative mood to a “STOP” signal. In the case of the present task, the natural tendency would correspond to the global processing (see Huntsinger et al., 2010, for a complementary discussion). Mood, as a dynamic affective state, acts as a coordinative contextual factor for visual perception in humans that eventually determines how visual detectors are oriented, and what is actually seen.

### THE EXPERT EYE MOVEMENT AND COGNITION-PERCEPTION COUPLING

The orientation of the eyes is one of the earliest stages of vision organization, on which contextual non-visual parameters can intervene. In the trend of expertise research, several studies have shown that (i) experts deploy their eye movements differently from more novice individuals, in their domain of expertise, and (ii) the nature of cognitive expertise is coupled with the type of visual search that is employed. Reingold et al. (2001) conducted research on chess expertise and eye movement. They demonstrated in a check-detection task that experts have a greater visual span for structured configurations of games. Chess experts extracted information from both foveal and parafoveal regions and were able to process interpiece relations. They produced a fewer number of fixations per trial than other participants. The other interesting and synergic point is that experts had a higher proportion of eye fixations that fell between individual pieces rather than on the pieces. Those data on the larger visual span in experts were congruent with others collected in a change detection task, combining the flicker and the gaze-contingent window paradigms. Expertise decreased change blindness, but only when patterns where structured and corresponded to real, possible game situations. Taken together, these results clearly indicate that visual search strategies in experts are different from those found in novices or in intermediates. The expertise of an individual heavily constrains what he looks at and how wide his visual span can be.

In the same vein, Laurent et al. (2006) reported data on visual search strategies in expert and novice basketball players. Participants performed a same-different judgment task of schematic basketball scenes pairs. Participants were asked to decide whether the two scenes – that were sequentially presented – were identical or different. Differences were local distortions of the position of zero, one, two, or three player(s) on the playground. Results showed that experts made fewer errors than novices and that visual search in expert basketball players was poorly sensitive to local distortions in contrast to what was found in novices, where the number of eye fixations on the second configuration was linearly and negatively correlated to the number of local elements that were displaced. Therefore, it seems that experts have a consistent and rather relation-oriented visual search, whereas novices have an attribute-oriented visual search.

All these data are important to the present development on context-sensitive vision, since eye-tracking research in the field of expertise is congruent with former studies on expertise focusing on higher-level mnemonic processes. The latter already showed that experts had a superior ability in recalling or recognizing structured patterns of game. The coordinative nature between cognitive and lower-level perceptual processes has already been developed elsewhere (Laurent and Ripoll, 2009). Through some processes such as categorical perception, perception becomes attuned to critical visual features that are diagnostic of higher-level successful categorizations (Goldstone, 1994; Schyns et al., 1998). Therefore, cognition and perception become coordinated, not only because cognition exploits perceptual information, but also, because vision becomes coordinated with the needs of cognitive activity. The coordination suggests that cognition is also, as it is the case of other processes developed in the preceding paragraphs, a dimension of the ecology of vision.

Data belonging to this (non-comprehensive) review on the influences of non-visual processes over vision serve to illustrate the recent empirical endeavors that should be taken into account in order to discuss the classical view of cognition as a set of process restricted to a “sandwiched” layer *between* sensory inputs and motor outputs, (see also, for such a critical discussion of the classical view; Varela et al., 1991; Stewart et al., 2010). Note that in this classical view, sensory and motor processes are rarely regarded as “cognitive.” In opposition to those postulates, perception and action, in this article, are regarded as *basically coupled with the organism goals* or, to avoid confusions about any hypothesized abstraction level of “goals” in organisms, in other words, the teleological dimensions of the organism subsystems. All the dimensions of the human being that represent some pressure in order to achieve the equilibrium of the organism and to change its internal state or the state of its coordination with the environment (e.g., thirst, emotions, higher-level motivations) embody the teleology.

So far, I have addressed arguments that should help us refine our understanding of the nature of vision. Vision, though often regarded as “hard-wired,” essentially bottom-up, is, as other “later” cognitive processes, embedded. Visual perception and behaviors emerge from complex interplays between different biological, psychological, and social dimensions. Modeling variance of behaviors and phenomenal processes implies to put these processes into their context. One major obstacle in this perspective lies in the increasing analytical decomposition of research objects, as well as probably in the institutionalization of disciplines and scientific careers involving specific objects and methods that might be too narrowly defined. Therefore, psychological concepts usually tend to get proximal explanations in the primary field of expertise of researchers. However, as demonstrated just above, many dimensions influence a given psychological process; and the lack of conceptual integration decreases the potentials for a synthesis. This synthesis is needed in order to get a unified approach to human psychology, and to prepare future psychologists to the diversity of dimensions they could usefully model and modify in the complex real world.

In order to get further into the formalization of the multiple couplings involving different scale levels, I will discuss recent biocomputational developments. These developments may have non-marginal influences over our quantitative representations of networks, connectedness, and their transformation with time, and could help us refine our representation of the coordination between processes and scales of analysis.

## ZOOM IN! BIOCOMPUTATIONAL FOUNDATIONS OF MEM APPLIED TO VISION

### STRUCTURAL AND FUNCTIONAL CONNECTIVITY: BASIC PROPERTIES FOR CONTEXTUAL INFLUENCES IN THE BRAIN

The dynamic processes that have been described so far at the psychological level are specific cases of complex interactions. Properties of complex networks have been studied in biological and computational neuroscience (Scannell and Young, 1993; Scannell et al., 1995, 1999; Sporns, 2011). The complexity of networks, defined as interconnected nodes, lies in their size, and in the interaction between network’s architecture and dynamics (Sporns et al., 2004). Both the behavior of individual nodes and the architecture of their interconnections give rise to a global equilibrium and to behaviors. A major conclusion of these analyses is that the brain is a highly integrative system (Tononi et al., 1998; Tononi, 2004). Full delineation of brain structural and functional connections is currently an object of effort for a group of scientists who work on the definition of “human connectome” (Sporns, 2011, 2013; Smith et al., 2013; Van Essen, 2013; Van Essen et al., 2013).

At the structural level, data coming from biological and computational neurosciences, neurology and neuropsychology not only revealed specific roles attached to brain areas in the process of vision (Zeki et al., 1991; Tootell et al., 1998; Rolls and Deco, 2002), but have also recently shed light on the “anatomical hubs” present in some brain regions (He et al., 2007; van den Heuvel and Sporns, 2011, 2013), among which we find parts of the visual cortex. He et al. (2007) founded their works on the analysis of covariation in cortical thickness, because they assumed that covariation could be due to “mutually trophic” influences. These covariations were previously found to associate visual cortex, lateral geniculate nucleus, and optic tract (Andrews et al., 1997), a network critical to the emergence of visual representations. Based on this methodology, He et al. (2007) revealed that brain networks are made of “small worlds,” short- and long-range connections usually found when human diffusion imaging is used. The structural architecture of neural networks seems to be highly related to functional activity and information exchange through neural nets. Some diseases such as some forms of Alzheimer disease (AD) provoke a disruption in neural pathways, including visual system’s networks. This can occur from the primary visual cortex level to visual associative areas (Morrison et al., 1991). In those cases, impairment at the structural anatomical level has direct consequences upon functional connectivity: elementary integration of visual information is weakened.

At the functional level, studies have been developed in order to account for complex biophysical coordinations between brain areas (Varela et al., 2001), or even between different brains (Dumas et al., 2012). Rodriguez et al. (1999) studied how the synchronization of oscillating neuronal spikes occurs in the brain in the frequency range 30–80 Hz (i.e., gamma oscillations). In their experiment, participants viewed ambiguous stimuli, which could be perceived either as faces or as meaningless stimulation. Participants were asked to indicate whether they perceived a face or a meaningless stimulation by pressing one of two answer keys. Results showed that visual perception of faces but not meaningless stimulation corresponded to periods of “long-distance pattern of synchronization” in the gamma band between the left parieto-occipital areas and frontotemporal regions. In this case, the coupling between occipital (massively involved in the visual processing of retinal information), parietal (spatial cognition and episodic memory), and fronto-temporal (recognition and perceptual learning) regions was conceived as the neural bases for perception. The principle of *co-increasing* in activation (measured for instance by the regional cerebral blood flow) in infero-temporal and occipital areas has been confirmed later in brain imaging protocols, as being a basic neural process underlying visual categorical perception of face familiarity (Rossion et al., 2001) and other emotional influences on perception (Adolphs, 2004; Vuilleumier and Huang, 2009). Rodriguez et al. (1999) pioneering electrophysiological study identified electrophysiolocal markers of brain coordination during visual perception process. After the perception phase but before the motor one, that is during the transition between perception and action, a desynchronization was reported. Finally, when the participants were launching the motor response, a new phase of synchronization (involving slightly similar couplings between right temporal and central regions in both meaningfulness conditions) was reported. This research has shown how each phenomenological (through the analysis of the perceptual phase), or behavioral (through the analysis of the motor phase) activity, in this task, was embodied in specific couplings and decoupling between neural nets. Based on a similar electrophysiological analysis, Varela et al. (2001) have proposed a framework for conceiving the unity of some “cognitive moments” and the distributed architecture of neuroanatomy. Coherent behavior and cognition would emerge from large-scale integration. The underlying mechanisms would consist in the synchronization of electrical activities “over multiple frequency bands.” Varela et al. (2001) stressed the reciprocal nature of connections in the process of integration, in opposition to the more strictly bottom-up view of integration, in which the process is conceived as the computations carried out between sensory and motor areas (i.e., in “associative” areas).

More recently, Vuilleumier and Driver (2007) described the evolution in the use of fMRI for characterizing brain contribution to a given function. If early use of fMRI consisted in attributing functions to particular brain regions or networks, advanced research in the domain is currently investigating how distant regions in the brain interact with each other and change their own activity as a function of their current connectedness to other active regions. This trend is known as “functional integration.” Advances in the understanding of human brain provide evidence for complex coordination between brain regions. “*Functional connectivity arises from context-sensitive dynamics that unfold rapidly but are shaped by a backbone of structural connectivity that can change only slowly*” (Kleinschmidt and Vuilleumier, 2013, p. 333). Even if bottom-up models of vision have had a considerable influence over the way psychologists and neuroscientists have represented computation in visual systems (Marr, 1982), recent neuroimagery studies provide evidence for visual cortex activity modulation as a function of the connections between functionally related territories.

Nir et al. (2006) found a correlation between spontaneous fluctuations in blood-oxygen-level–dependent (BOLD) signals related to cortical regions – all involved in visual processing – in participants placed in the darkness (i.e., no light illuminating the scanning room). When participants were visually stimulated those correlational patterns suddenly changed. Cortical regions (e.g., left parahippocampal place area, posterior fusiform gyrus, superior temporal sulcus, post-central sulcus, central sulcus, lateral sulcus) exhibited large-scale synchronized slow (<1 Hz) fluctuations at rest. Coordinative patterns are changed as a function of contextual influences. For any region, the latter are represented by the activity of other connected cortical networks. When no retinal information is received, some brain regions involved in visual processing tend to synchronize their activity. In other words, not only does the activation pattern depend on external stimulation, but this pattern is also, at each point of the neural network, dependent on its structural and functional relationships to other parts of the network.

Vuilleumier and his colleagues have carried out a series of studies showing that emotions and other higher cortical controls involved in “attentional modulation” interact with vision at the cerebral level (Vuilleumier et al., 2001, 2004; Mazzola et al., 2013; see also Vuilleumier, 2005; Vuilleumier and Driver, 2007; Vuilleumier and Huang, 2009). They have been interested in long-range couplings between the “visual” cortices and distant regions that have traditionally been investigated separately (i.e., amygdala, fronto-parietal cortex). The visual effects of emotion, through the contribution of amygdala, and the visual effects of what the authors call “attention,” through the contribution of fronto-parietal circuits, have similar boosting effects on the activity of the visual cortex. They both contribute to enhance the processing of visual stimulus.

Similarly to what has been described as contextual effects at the behavioral level earlier in this paper, “*contextual” influences* occur almost everywhere in the brain. The highly converging nets create the biological basis for multiple couplings at different scales from the microscale of synaptic connections to the macroscale of area coordinations. The analysis of the coupling between brain areas, employed in the works reported in this section, offers exciting perspectives and should be one key element of a complex approach to vision in the future.

In order to improve the synthesis between brain, behavioral, and mind sciences, it would be very helpful to connect those studies involving vision with two important lines of research. The first line is constituted by the synergetic trend (Haken et al., 1985; Kelso, 1995, 2012; Kelso and Engstrøm, 2006; Kelso et al., 2013; Tomasi et al., 2014), which is rarely applied to the field of vision *per se*. This trend successfully gathers different components and analyzes their collective behavior through the tracking of order parameters, which characterize their mutual relations (e.g., phase relations of oscillators). This framework provides models of brain activity and behavior, by integrating the coordination of system components. The second line has involved research carried out by Menon (2011) and colleagues on the dynamic changes observed in the coupling between brain components. The research has sought to characterize psychiatric and neurological disorders. Menon conceives three major types of functional networks and relates diseases to specific alterations of these networks. Mechanisms underlying the pathological alteration of information processing are contextualized at different spatial scales, from abnormal small-world architecture, to large-scale functional disconnection. His approach does not involve the visual function, but gives interesting lends for conceiving different types of network evolutions and different consequences on the emergence of behaviors.

Despite the obvious intellectual contribution of the research mentioned up to this point, two questions are still pending. The first is related to how *different scale levels* of organization of the visual system are coordinated; the second concerns the characterization of the *meaning of subsystem behavioral changes*: in other words, why does a given component exert some specific constraints at some times, and others later?

### TOWARD BRIDGES BETWEEN SCALES OF ANALYSIS FOR A SYNTHESIS IN “VISUAL” BRAIN CONNECTIVITY SCIENCE

As previously put forward by the 1998 Nobel Prize winner in physics Laughlin (2005) “*reliable cause-and-effect relationships in the natural world have something to tell us about ourselves, in that they owe their reliability to principles of organization rather than microscopic rules*.” However, if organization is central to our issue, this takes place at different scales in the brain. Any synthesis (which involves connecting things, ideas…) endeavor in the field of mind, brain, and behavior must account for this multi-layered organization, within brain, and from brain to behavior.

Evidence for the emergence of neural and behavioral activities from multiple couplings at different scale levels can be found in studies of diseases affecting the central nervous system. Not only do the couplings occur at multiple scales in the brain, but also couplings occurring at different scales influence each other. For instance, multiple sclerosis has recently been considered as a disconnecting syndrome (He et al., 2009; Kleinschmidt and Vuilleumier, 2013), in which increased functional connectivity can be recorded. Though the impairment level of neural structures in various diseases is often negatively linked to the functional connectivity level assessed by fMRI (Yu et al., 2008; Zhou et al., 2013), the impairment of small-word neural nets in multiple sclerosis can lead to increasing the activity level of *larger nets* involving more distant regions within the brain (Kleinschmidt and Vuilleumier, 2013). At this point, we realize that the increase in functional activity at a scale level can be interpreted only if we analyze and understand what happens at *another scale level* in the brain. *The synthesis, the access to the global meaning of the system behavior emerges from the understanding of structures and events at different scales and from the conceptual relationship that is established between them*.

Vision heavily relies on network functionality *and* dynamics. Functionality is not over when partial impairments of local networks are found, because dynamics is possible. Different couplings at similar or other levels can compensate for the damage of local networks. This kind of neural plasticity involving compensatory processes that encompass different interactive scale levels (partially) supports the continuity of behavioral performance and phenomenological production. Further evidence for changes in the scale level of neural ensembles involved in vision has been reported in deaf cats (Lomber et al., 2010). In these animals, which exhibit supranormal visual performance (e.g., improved peripheral target localization; lower thresholds of movement detection) the “auditory cortex” is recruited during visual perception. Reversible deactivations of posterior auditory cortex and dorsal auditory cortex respectively suppressed any superiority in localization and in movement detection. Therefore, scale-dependent reorganizations involving the addition of neural territories occur in the brain to support heightened functional performance at the visual behavioral level.

The subject of interactions between scale levels has been recently addressed by Kim et al. (2013), in the context of the connectome project. They categorized recent works in the domain into three classes: macro-scale (inter-regional connectivity), mesoscale (neuron level and its projections), and microscale (including all synaptic contacts). The authors presented details of most advanced methodologies for characterizing each scale. The subsequent problem lies in the integration of information across scales, which implies to get partial common frames of references from one scale to another. This obviously technical issue lets basic questions concerning the nature of causal links between scales unanswered. At different levels of scientific analysis, progress in the description of networks is made in spite of material challenges. However, a model of couplings between neural activities belonging to the three scales is still lacking.

Our review makes it salient that data have been accumulated, which show that information (defined electrically or symbolically) and behavior are fundamentally dependent on the context. The latter emerges in the brain through structural and functional connections that constitute the background for potential coordinative patterns. Synthesizing involves gathering sparse arguments and unconnected conceptual tools. We must consider conceptual connections between what occurs at brain, phenomenological, and behavioral levels. A major issue for the integration of knowledge, the connection of unconnected fields in science, is the understanding of the ends of the variations. In order to shift from an operative view on couplings and complexity to a more global perspective where meaning can be associated with complex “computation,” an approach combining the emergence conditions of mutual influences and the meaning of these influences should be privileged.

## MEM AND ITS DYNAMICAL ZOOM: TOWARD THE THEORETICAL INTEGRATION OF CONNECTEDNESS AND EMERGENT TELEOLOGY

A synthesis in the field of mind sciences not only requires to gather information at different scales of analysis but also to shed light on *why* different systems are coupled and *why* their coupling evolves with time. We need to think and model the “motivational” forces that draw different scales of the central nervous system as well as phenomenological states and behavior toward a given and momentary equilibrium. However, the teleological dimensions of cognition and brain dynamics are more rarely modeled. The emerging goals that are pursued by the individual can also (as connectedness) be described at different levels of abstraction (Laurent, 2003). From low-levels of cognitive control over goals to the abstract management of personal motivation, individual forces that drive cognition must be integrated, as connectedness, in a conceptual model of mind. If much work has been done over the two last decades in the field of complex neural networks, connectedness and complex cognition, the integration of teleology and motivation in those works is far to be achieved. In the following paragraphs, I am presenting two descriptions of MEM that account for the various effects reported earlier in this paper. The first one is developed with psychological concepts, whereas the second one is developed with biocomputational concepts. The goal of the procedure is to provide supplementary opportunities for exchanges between disciplines. In both descriptions, the role of scale interaction is being discussed.

### DESCRIPTION OF MEM AT THE PSYCHOLOGICAL LEVEL

Once the theoretical framework – for (i) conceiving the coupling between a cognitive component and a larger whole, (ii) identifying the teleological dimensions emerging from the multiple components – has been developed, methodological tools should be coined in order to describe the macroscopic behavior of the system. For instance, for vision, the collective behavior may be tracked by the use of eye movement recording. This technique informs us about the parts of the environment with which a human subject interacts. This interaction is the macroscopic behavior emerging from a complex coupling between the visuomotor subsystem and any other subsystem that is connected to the visual system. According to MEM, the amount of visual modulation by other subsystems depends on both the structural and the functional connectivity. Structural connectivity is generated both innately and as a result of individual goals and prolonged experiences with environmental structures. This can be altered also by aging and disease. Functional connectivity is influenced by dynamic teleological pressure coming from the rest of the organism. This pressure can be transmitted through the structural connection within electrical and chemical systems. Knowing the structure of the visual system is useful but not sufficient in order to predict its behavior. We must *re-build the ecology* (both within the body and outside the body) in order to conceive visual-motor dynamics. What the individual searches for in the environment is dependent on his emergent “motivation.” Here, “motivation” is wholly embodied and distributed in all the living system, each component (as shown previously in the empirical part) exerting a pressure on what visual search and experience finally are. The variance in eye movements, unexplained by a single factor, can be further investigated in the framework of multiscale couplings.

**FIGURE 1:**
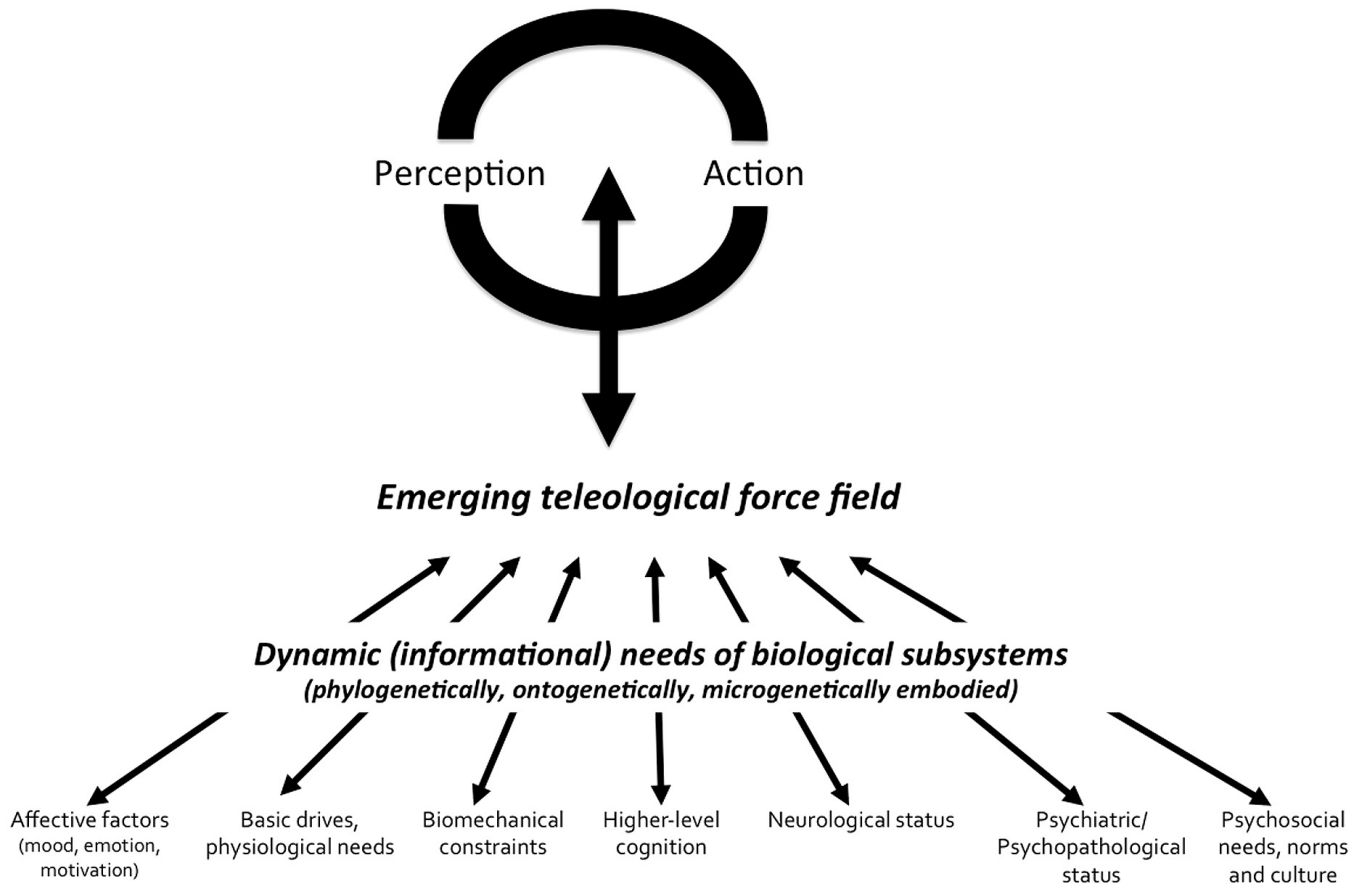
**Schematic description of MEM at the psychological level**.

**Figure 1** presents a summary of MEM organization at the *psychological level*. This lets us draw perspectives for further empirical research within a complex approach to perception. The influence of teleological factors is embodied in perceptual and motor activities, which are not “encapsulated,” but rather penetrated by non-perceptual or non-motor factors with which they form systems at different scales, that is, from the neurological to the social-cultural scale. The conceptual synthesis in this model lies in giving an account for evolving (and interacting) ends at different levels of abstraction as well as predicting their effects on cognitive systems. In other words, this consists in considering cognitive systems as essentially *goal-driven*, in recognizing that what is processed fundamentally depends on the current characteristics of all nets that are connected to them. The *synthesis* between disciplines is only possible if researchers are able to recognize not only that their research object is influenced by various factors pertaining to multiple and flexible scales of abstraction, but also if they systematically engage in the description of the motivations or teleology attached to these factors. In this framework, *context-sensitivity* is a basis for the conception of open systems and the intellectual synthesis between classically separated research fields. *The focus of MEM is both on multiscale connectedness (or functional coupling) and embodied teleology (or distributed and local autopoiesis)*. Connectedness is often cited as a critical factor of system dynamics, but the scale at which this is considered is rarely flexible. Teleology is very rarely evoked. Nonetheless, as reported in the precedent sections of this paper, the change in a subsystem “needs” dramatically modifies the strength of the influence this subsystem will exert on visual search for information and attunement to features that are critical at any given time. Taking into account these needs and their connections with visual and mental processes allows us modeling multiscale environments of psychological processes, and then contributing to rebuild the *ecology of mind*.

The perception-action cycles are under the influence of multiple embodied motivational forces (i.e., the emerging teleological force field). The notion of “emerging teleological force field” refers to the sum of electrical and chemical information emerging from local neural nets and single neurons, which translate local constraints and needs into constraints for search of information, through patterns of functional neural connections. The multiscale enaction lies in the following principles: (i) any part of the body plays a role in the definition of the search needs for information and operational closure of the organism (ii) the strength of local influences over perception-action cycles is influenced by factors whose expression duration ranges from macrogenetic (i.e., evolutionary) to microgenetic (e.g., glycemic regulation needs) scales (iii) perception-action cycles are interfaced with different needs, which can be associated with various levels of abstraction (iv) the autonomous activity produced at any cellular unit or ensemble of cell is an expression of the autopoietic characteristic (i.e., affirmation of self-identity) of living units, so that cooperation and competition are basic properties of relations between cells or groups of cells.

A (visual) system able to take into account the initial conditions of the agent is a real intelligent system, since this one has an immediate adaptation to the *dynamic* or *evolving goals* of the agent in which it is embedded. Therefore, understanding how different cognitive or neural systems interact becomes central to theorizing the variance of visual perception. Furthermore, each identified interaction between visual and non-visual processes becomes a new opportunity of understanding and influencing world representations and behavior. The proposed conceptual model is empirically testable. Each of the connections represented in the **Figure 1** has already been exemplified and experimentally tested, and supporting data have been reported in our review. However, its heuristic value is also related to the acknowledgment that visual and motor systems are influenced by other components that provide them with teleological perspectives. *The connection between biological-cognitive processes on the one hand, and their meanings in a wide range of coupling contexts on the other hand*, is fundamental to the integrative science of complex living systems. MEM is, for those reasons, in part in rupture with Maturana and Varela (1980) assumptions according to which autopoietic systems are strictly non-teleological. These pioneering authors had probably to distinguish between classical functionalism and teleology, on one side, and their autopoietic systems theory, on the other side. This was aimed at eliminating any reference to an abstract “purpose” that would, from the external world, guide the regulation of behavior. In this sense, I agree with Maturana and Varela’s (1980) project to naturalize our theories of human cognition. There is no need to evoke any external law to the human subject in order to found our understanding of individual regulation. This is not that external constraints do not influence individual behavior, but rather that any meaning arises in the context of self-referenced evaluative processes and values. However, with MEM, I propose to stress the *flexibility* of what context is and what the autopoietic levels should be in *both* our theoretical *and* empirical research. In MEM, there are multilayered recursive loops and identity-affirming processes. The whole body is not the only autopoietic unity; at another end of the continuum, the single cell has also its own organization. Signals processed by the cells generate recursive loops. Neural ensembles behave through excitatory or inhibitory activity, and influence perceptual-motor systems. In this perspective, perceptual-motor activity in frontal, parietal, temporal, and occipital areas embodies goals, needs, emerging in distal regions. In this sense, the ends of perceptual-motor systems are strictly emerging, by summing local action potentials. Once again, the teleology mentioned here is in no way similar to an external principle that would indicate, from outside the organism, the ends of a living being, specie, or an individual. Well before biologists, psychologists or cyberneticists, Nietzsche already warned his readers against “teleological” discourses.

*“There is no denying that* in the long run* each of these great teachers of a purpose was vanquished by laughter, reason and nature: the brief tragedy always changed and returned into the eternal comedy of existence, and the ‘waves of uncountable laughter’ – to cite Aeschylus – must in the end also come crashing down on the greatest of these tragedians.”*

*Friedrich Wilhelm Nietzsche, 1882.* The Gay Science. * Book One, 1, “The teachers of the purpose of existence.”*

Instead of explaining the regulation of behaviors or existence in relation to *external* causes, or in relation to elementary “cognitive functions” that are usually thought of as if the specific context of their evaluation did not count, I suggest to consider humans as being made of contexts (subsystems), many of which signal the critical environmental parts to deal with as a function of the autopoietic necessities of the moment. The ability of these subsystems to orient perceptual-motor cycles can be referred to as emerging teleological constraints. These teleological constraints need not be symbolically represented. They are embodied through metabolic and electrical activity, which take their meaning in relation to local basic autopoietic units that are the cells.

### DESCRIPTION OF MEM AT THE BIOCOMPUTATIONAL LEVEL

As proposed by Capra and Luisi (2014, p. 155), “[e]mergent properties are the novel properties that arise when a higher level of complexity is reached by putting together components of lower complexity. The properties are novel in the sense that they are not present in the parts: they *emerge* from the specific relationships and interactions among the parts in the organized ensemble.” *MEM applies this principle to teleology*. In other words, the global goals that are pursued by the individual emerge from the coordination between multiple more elementary teleological forces at the subsystem level. In the present paper, I reviewed the role of basic needs, mood regulation goals, social-economical determinants. All these “local” parameters are forces that are progressively and hierarchically coupled to cognitive and perceptual processes.

The modalities of local needs combination and integration should receive computational solutions. Systematic research has to be carried out in order to reveal how vision emerges as a consequence of information integration. For instance, careful control over needs or goals should be observed in order to measure the effects of the strongest needs and goals of the wider agent system at any given time, in comparison with less demanding ones. A simple win-loose principle can be envisaged in order to perfectly satisfy the most valued goal, but complex motivations could also lead to a multipurpose visual search connecting different goals with vision. At a more elementary scale, MEM is characterized by connectedness and informational exchanges between the organism subsystems. The roles and positions in space of these subsystems are various. In order to account for the perceptual-motor dynamics occurring under the influence of multiscale information integration, a hierarchical process taking into account interactions between different parts of the system will be proposed. Contemporary advances of theoretical biology can be considered as foundations for some of these computations carried out by MEM. Chauvet (1993a,b,c, 2004) proposed mathematical tools accounting for the hierarchical information integration. In this framework, information integration is under the constraints of several organizational factors associated to biological systems. According to Chauvet (2004, p. 211), “the functional organization of a biological system can be represented by a mathematical graph in which the summits correspond to the sources and sinks of the system and the arcs correspond to functional interactions between them.” Chauvet takes space and time into account. He introduces the concept of *structural discontinuity* in order to account for the propagation of functional interaction through different milieux. If there is a discontinuity between the source and the sink, the interaction Ψ will need to go through the inferior level ϕ. Chauvet proposes the *S*-propagator (*Structure*-propagator) notion, which is an operator describing the move of activation through structural hierarchy. The *S-*propagator corresponds to the following mathematical function:

$$P_{i0}=P_{0}\Phi P_{i}\,\;\;\;\;\;\;\;\;\;\;\;\;\;\;\;\;\;\;\;\;\;\;\;\;\;\;\;\;\;\;\;\;\;\;\;\;\;\;\;\;\left(2\right)$$

where the propagation between two discontinuous spaces, the source situated at *r_i_* and the sink situated at *r_0_*, involves the interaction ϕ from which it results. According to Chauvet (2004, p. 227), three steps are required in order to propagate the functional interaction Ψ between different structures:

(1) The propagation of the functional interaction within the source, involving the operator *P*_i_.
(2) The modification of the interaction by the structural discontinuity generating another interaction at a lower level ϕ.
(3) The propagation of the Ψ interaction at the higher level in the sink at *r*_0_.

**FIGURE 2:**
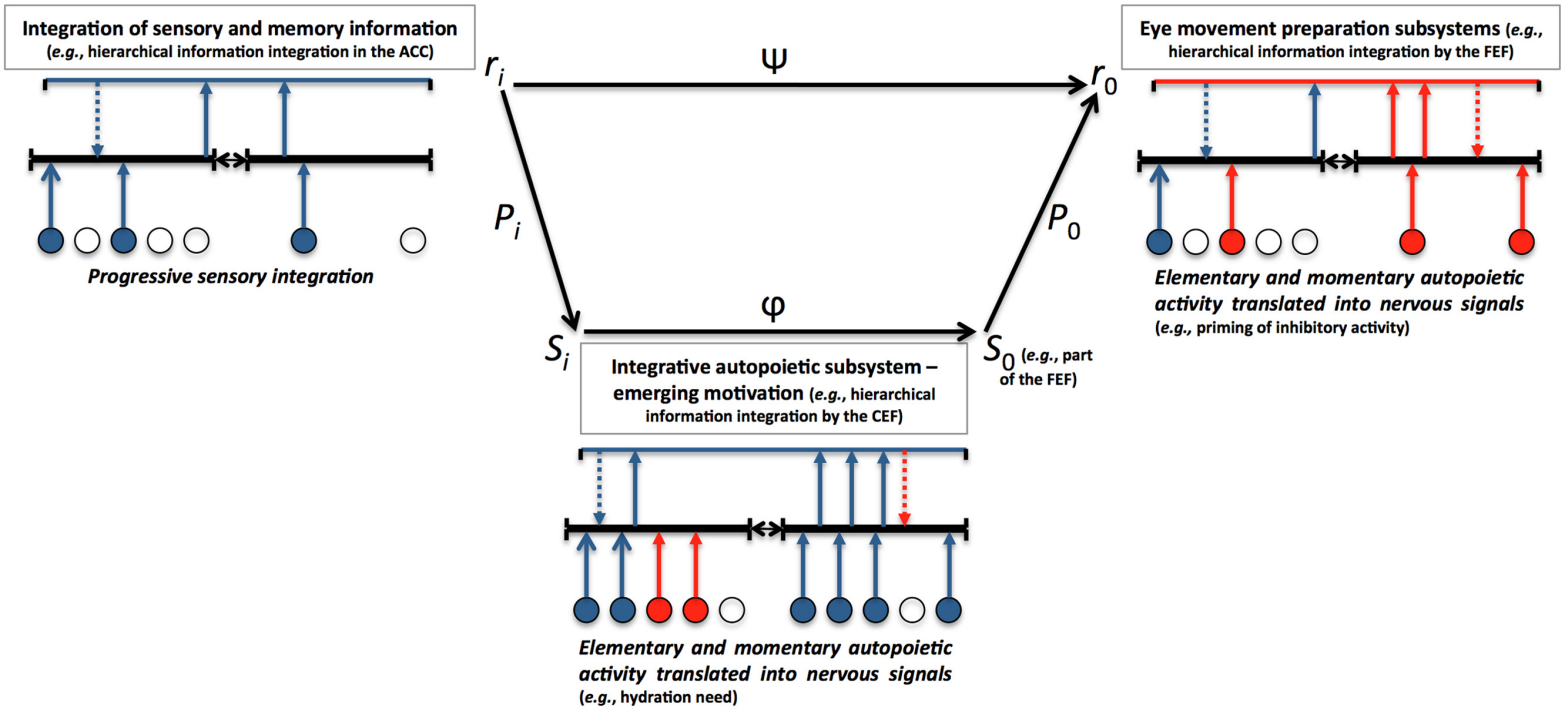
**Biocomputational solutions to multiscale enaction in MEM**.

Between distant interacting structures, other lower-level dynamics moderate information transmission. **Figure 2** illustrates how MEM integrates those principles of theoretical biology and relates elementary autopoietic activity to structural organization and emergence of eye movement control and visual search of information. Each structural space is associated with a dynamics that contributes to shaping upcoming and downcoming information, such that the communication between two structures depends on both (lower-lever) elementary autopoietic dynamics and (higher-level) integrative dynamics.

In this example, the functional interaction between the anterior cingulate cortex (ACC) and the frontal eye field (FEF) is noted Ψ and is allowed by the propagation of the interaction in the source *P*_i_ toward the inferior dimensional level *S* (in the cingulate eye field [CEF]). The propagation of the interaction ϕ at the *S* level is shaped by the dynamics of the CEF, such that the functional interaction at the Ψ level is dependent on the upcoming *P*_0_ interaction. Beyond those solutions inspired by theoretical biology (Chauvet, 2004), MEM relies on a principle of multiscale and emerging autopoiesis from the cellular level to the most integrative structure that is the organism. Instances of potential activation patterns are presented. The presence [arrow]/absence [no arrow] of cell excitatory [blue]/inhibitory [red] signals, is a function of both *local cell needs* and* structural activity*. Dashed arrows represent top-down connections and potential modulation of upcoming information by higher-level neural nets. The integrative autopoietic subsystem acts as a filter of the interaction propagation between the ACC and the FEF, and contributes to influence eye movement and search for information.

Emerging motivational information space can be conceived as a fundamental integrative autopoietic field, beside the more elementary manifestation of autopoiesis at any cellular level of the organism. Structural discontinuity between perception-memory and action can be filled in by propagating functional interaction through the motivational subsystem.

Multiscale Enaction Model conceives enaction as the result of multiscale integration, self-affirming of identity, and meaning emergence, *at different structural levels*. In other words, if MEM contributes to gather processes classically associated with different scientific disciplines, it also recognizes the multiplicity of the human being and, at the psychological level, the role of *emerging* teleologies in the constitution of the perceptual-motor *Umwelt*, and then, of the subjective world. As pointed out in the elegant and huge work by Thompson (2010, p. 14): “[o]ne key point is that the enactive approach explicates selfhood and subjectivity from the ground up by accounting for the autonomy proper to living and cognitive beings.” It is required, at this time of our scientific developments to better understand, and further research – both theoretically and empirically – the process by which, a kind of operational closure occurs through perceptual and motor processes. However, in contrast with some “enactive approaches” that reduce the causes of emergence to sensorimotor activity, MEM includes multiple scales of determinants, ranging from cellular autopoiesis to complex embodied social behaviors. The embodiment process of motivation lies in the *progressive hierarchical integration* of local “needs.” MEM connects embodied motivation to visual search behaviors, perception, and the subsequently emerging subjective world.

## CONCLUSION

In this article, I have proposed a model (MEM), which integrates complex and dynamical approaches to vision, on the basis of experimental results. This approach highlights the need to consider both the connective characteristics of visual hardware, and the dynamic nature of the coupling between various bottom-up and top-down processes. The enactive multiscale view that has been proposed, in the continuation of earlier enactive proposals (Varela et al., 1991; Thompson, 2010), is based on empirical data showing that human visual-perceptual activity is strongly dependent on non-visual and non-motor activity. This implies to contextualize vision in the more global framework of functional hierarchical systems (Proffitt, 1993) that are dynamically shaped by the current *embodied* needs of the system. This differentiates the current proposal from previous enactive works more strictly based on sensorimotor loops and contingencies. As dynamical systems, humans are made of evolving needs (e.g., satisfying thirst, collecting specific information, reaching a place in the physical environment), and then what they search for in the environment changes with time as a function of internal initial conditions. Those initial conditions conduct the human organism to *enact* a given world.

The study of coordination between visual and non-visual factors makes it urgent to develop “an embodied motivational psychology of perception” (i.e., not confined to abstract views on elaborated or symbolically driven motivations, but based on any teleological perspective embodied in the organism). The enactive approach proposed here tries to capture sources of variability in perception. In order to achieve our goals of understanding finalized vision, we must understand the very nature of the respective activities of the systems that are found to be coordinated with vision, as well as the modes of coordination that are variously employed by the organism as a function of time.

Conceiving artificial agents whose properties would be inspired from those fundamental principles may also contribute to reduce the gap between what is known about “connected cognition” in humans and cold or “disconnected cognition” in some machines (see Vunka Jungum and Laurent, 2009, for a discussion of this problem in the context of emotions; and Arkin, 2005, for some lends concerning the inclusion of emotion-related processes in robots). Given processing time- and power-limitation of human cognition (i.e., bounded rationality; Simon, 1956, 1957; Gigerenzer and Selten, 2002; Kahneman, 2003; Hertwig et al., 2013), the modeling of couplings between vision and needs/current priorities may well be a strong foundation for the understanding of true intelligent behaviors.

Finally, each revealed influence of non-perceptual factors on perception also offers the opportunity of psychological intervention in order to change psychological equilibrium. Empathy, in the framework of psychological science, is not only a process of goodwill and compassionate listening, but also implies to understand the context in which the individual is embedded. The kind of approach to perception developed here should facilitate the meeting between real-world complexity and the theorization of human mind and behavior. Any synthesis in the field of mind, brain, and behavior, implies to conceive complexity, which is the quality of a system made of multiple entities that are related to each other by multiple connections. Conceiving complexity at different levels of abstraction also requires the openness of research objects and systems in order to coordinate scientific concepts. This kind of synthesis should not only be beneficial to scientists, but also to professional intervention in the field of psychology. Psychologists and human-centered professionals have not psychic, biological, cognitive, or social beings to deal with. They face whole complex systems whose behaviors emerge from the multiple couplings between various determinants. Constituting interactions in perception as a privileged object of psychological science should also allow reducing the frustration of students and professional when they shift from theory to complex and real settings on the field and they establish that the whole is greater than the sum of the parts. Perception is an early process. Getting tools in order to manipulate this primitive cognition should be an asset to change the *Umwelt* of humans, that is, the perception of what the world is and what the possible actions in the world are.

To summarize, following the presentation of MEM applied to complexity in vision, the best way to contribute to a modern synthesis in the field of mind sciences is to develop and employ a framework based on the recognition of multiscale couplings. The latter result in the emergence of multiple embodied teleological forces (i.e., dynamic patterns of electrical and metabolic activity), which induces – at each moment – a specific operational closure with the environment. In other words, we get perceptually- and motor-tuned to our local and global emerging needs. Not only does this model contribute to reuniting body, mind, and behavior, but it also achieves thematic and subdisciplinary connections within psychology, by connecting objects that are usually researched independently as “functions” and that have progressively acquired both modularity and functionality statuses, because of the lack of theoretical, methodological, and empirical integration (or contextual relativism).

## Conflict of Interest Statement

The author declares that the research was conducted in the absence of any commercial or financial relationships that could be construed as a potential conflict of interest.

## Footnotes

1 This quote is provided in English and discussed in the context of my theoretical proposal, later in the text.
